# The WHO and UNICEF Joint Monitoring Programme (JMP) Indicators for Water Supply, Sanitation and Hygiene and Their Association with Linear Growth in Children 6 to 23 Months in East Africa

**DOI:** 10.3390/ijerph17176262

**Published:** 2020-08-28

**Authors:** Hasina Rakotomanana, Joel J. Komakech, Christine N. Walters, Barbara J. Stoecker

**Affiliations:** Department of Nutritional Sciences, Oklahoma State University, Stillwater, OK 74078, USA; joel.komakech@okstate.edu (J.J.K.); christine.n.walters@okstate.edu (C.N.W.); Barbara.Stoecker@okstate.edu (B.J.S.)

**Keywords:** water quality, sanitation, hygiene, stunting, East Africa

## Abstract

The slow decrease in child stunting rates in East Africa warrants further research to identify the influence of contributing factors such as water, sanitation, and hygiene (WASH). This study investigated the association between child length and WASH conditions using the recently revised WHO and UNICEF (United Nations Children’s Fund) Joint Monitoring Programme (JMP) indicators. Data from households with infants and young children aged 6–23 months from the Demographic and Health Surveys in Burundi, Ethiopia, Kenya, Malawi, Rwanda, Tanzania, Uganda, and Zambia were used. Associations for each country between WASH conditions and length-for-age z-scores (LAZ) were analyzed using linear regression. Stunting rates were high (>20%) reaching 45% in Burundi. At the time of the most recent Demographic and Health Survey (DHS), more than half of the households in most countries did not have basic or safely managed WASH indicators. Models predicted significantly higher LAZ for children living in households with safely managed drinking water compared to those living in households drinking from surface water in Kenya (β = 0.13, *p* < 0.01) and Tanzania (β = 0.08, *p* < 0.05) after adjustment with child, maternal, and household covariates. Children living in households with improved sanitation facilities not shared with other households were also taller than children living in households practicing open defecation in Ethiopia (β = 0.07, *p* < 0.01) and Tanzania (β = 0.08, *p* < 0.01) in the adjusted models. All countries need improved WASH conditions to reduce pathogen and helminth contamination. Targeting adherence to the highest JMP indicators would support efforts to reduce child stunting in East Africa.

## 1. Introduction

Poor growth during the first thousand days leads to negative consequences ranging from decreased immunity to reduced academic performance in adulthood if not corrected early [1]. During the past two decades, child undernutrition rates have declined considerably due to the prioritization of the nutrition agenda worldwide. More specifically, global stunting rates for children under five decreased from 32.6% to 22.2% between 2010 and 2017 [2]. However, the reduction in child stunting in sub-Saharan Africa remained slow from 46.1% in 1970 to 40% in 2010 compared to the global reduction rate of 25.1% [3]. Therefore, the persisting high rates of stunting continue to be a public health concern in sub-Saharan Africa. Stunting rates remain especially high in the East African region where 39% of the children under five were stunted based on data from 2010 to 2016 [4].

**Figure 1:**
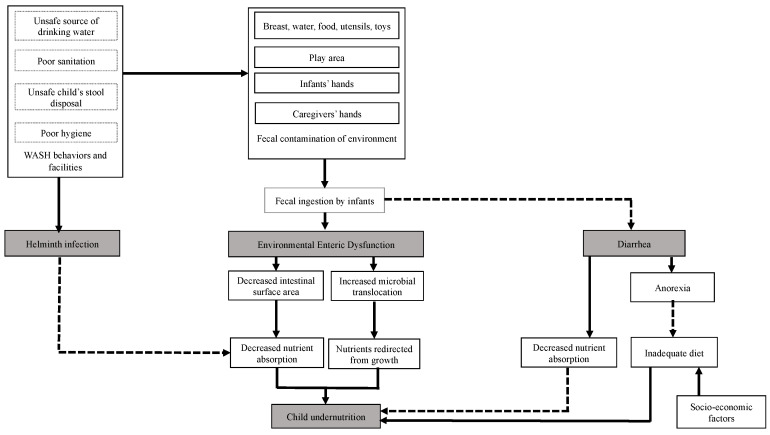
Pathways linking water, sanitation, and hygiene (WASH) conditions and child undernutrition. Adapted from Humphrey [15] and Dangour et al. [17]. Solid lines represent primary pathways and dotted lines represent secondary pathways.

Identifying determinants and addressing constraints related to child stunting are crucial in designing effective nutrition-specific and nutrition-sensitive interventions and policies needed for progress worldwide. Furthermore, the importance of the underlying factors of stunting may vary across countries and even across regions within a country [5]. Thus, further investigation of the contributing factors to malnutrition, such as inadequate water, sanitation, and hygiene (WASH) conditions and their role in child growth faltering is needed. Poor WASH practices have been associated with suboptimal child growth in sub-Saharan populations and globally [6,7,8]; and, improved WASH conditions have been accompanied by better child anthropometrics in several studies [6,7,9,10,11,12]. Figure 1 summarizes three pathways suggested to explain the role of unsafe WASH in child stunting. Repeated episodes of diarrhea and chronic helminth infections can reduce nutrient absorption leading to undernutrition [13,14]. Additionally, environmental enteric dysfunction (EED), widespread in areas with poor WASH indicators, has been suggested to cause impaired linear growth in children [15,16]. A pooled analysis from 140 countries reported that open defecation explained 54% of the height variation in children under the age of five [9]. Thus, inadequate WASH practices likely contribute to the slow reduction in child stunting rates in sub-Saharan Africa.

However, large cluster randomized trials recently reported no effect of individual or combined WASH interventions on linear growth in children under 5 years [18]. Moreover, compared to improved nutrition alone, there were no additional benefits of combining better WASH and nutrition on child length. Because of the high levels of pathogens reported from the children’s hands and in the drinking water, the low-cost and household-level WASH implemented in these studies may not have been sufficient to reduce pathogen contamination [18,19]. These results suggest that more elaborate and more specific WASH services may be needed to limit pathogen transmission from poor WASH practices. The UNICEF and WHO Joint Monitoring Programme for Water Supply, Sanitation and Hygiene (JMP) provides criteria for global standardized WASH indicators [20]. The indicators have been updated to include more detailed specifications, called “ladders”, within each category. These indicators can be used to monitor progress towards safe WASH conditions and in research analyses [20,21,22]. To our knowledge, there are limited studies examining links between WASH assessed with the newly refined JMP indicators and child stunting in the East African region.

To remedy the slow progress towards a reduction in child undernutrition in the region, further investigation of nutrition-sensitive as well as nutrition-specific factors contributing to the high prevalence of stunting in the East African region is critical. Analyzing the associations between the different levels of the JMP indicators and child length provides additional insights on the type of WASH interventions that may contribute most effectively to reducing stunting rates for each country. The purpose of this study was to evaluate the association between WASH conditions and child length in East Africa using nationally representative data. The results from this study can be used to adjust and prioritize future nutrition-sensitive policies and interventions to reduce childstunting in the region.

## 2. Methods

### 2.1. Study Population

The latest nationally representative Demographic and Health Survey (DHS) data from eight East African countries: Burundi (2016–2017), Ethiopia (2016), Kenya (2015), Malawi (2017), Rwanda (2017), Tanzania (2017), Uganda (2016), and Zambia (2013–2014) were analyzed. Children aged 6–23 months with available data including anthropometric measurements, WASH indicators and maternal and household characteristics were included in the analyses. Sample sizes included for each country are presented in Table 1. The DHS data collections were approved by ICF International’s Institutional Review Board and by each host country’s ethics committee. Secondary and de-identified data were used in this study.

### 2.2. Child Length

The primary outcome was child length-for-age z-scores (LAZ) based on the 2006 WHO Growth Standards [23].

### 2.3. WASH Indicators

The WASH indicators were based on the specifications of the WHO and UNICEF Joint Monitoring Programme for Water Supply, Sanitation and Hygiene (JMP) [20]. Details of each JMP ladder are summarized in Table 2. Each household was given a rank in ascending order from zero to four for water and for sanitation based on quality, access, and availability of the facilities or services. The safely managed water category did not include free of fecal and microbial contaminations as these data were not available. Not all of the households included in the surveys were asked questions regarding child or adult feces disposal; thus, the “safely managed” step of the sanitation ladder was excluded. The hygiene indicator was ranked from zero to two for each household. Higher indicator scores represent better WASH conditions.

### 2.4. Covariates

Known underlying factors affecting child linear growth [24] were included as covariates. Child characteristics included sex, age, and breastfeeding status. Maternal covariates included highest level of education, age, and height. Household covariates were wealth index and area (urban vs. rural) of residence.

### 2.5. Statistical Analyses

Weighted means and frequency distributions were used to describe the study population taking into consideration the clustering due to survey design. Indicators within each JMP ladder were treated as categorical variables. Linear regression models assessed the association between JMP WASH indicators and child LAZ for each country individually. First, unadjusted models were fitted (Model I). Then, models were adjusted for child, maternal, and household covariates in Model II. Additional adjustment with the other WASH indicators was conducted to establish the contribution of individual WASH indicators to child length in Model III. A significance level of 5% was set for the models. Households with missing values for WASH indicators were not included in the analyses. Statistical analyses were conducted with SAS 9.4 (SAS Institute, Cary, NC, USA). Because most of the households in Kenya, Rwanda, and Zambia were not asked questions related to handwashing, associations between hygiene and child length could not be modeled for those three countries.

## 3. Results

### 3.1. Characteristics of the Study Populations

Stunting rates were high (>20%) in all countries with the highest stunting rate in Burundi (45%) (Table 3). Maternal education levels were remarkably different across the eight countries. In Ethiopia, 60.5% of mothers had not received any formal education while this category was less than 12% in five of the eight countries. Ethiopia (35.2%) had the highest proportion of households in the lowest quintile of wealth index while Tanzania had only 14.4% in the lowest quintile. Lastly, most of the households surveyed across countries lived in rural areas.

In all countries except Burundi and Kenya, more than half of the population were classified as not having access to basic or safely managed drinking water sources according to the JMP standards (Table 3). Kenya had the highest proportion of the population with safely managed water sources (32.6%) and Rwanda had the lowest (9.6%). Open defecation was common in Ethiopia (36.4%) but low in Burundi (2.2%) and Rwanda (3.5%). More than half of the households in Burundi, Malawi and Rwanda had improved sanitation facilities compared to only 9% in Ethiopia.

Ethiopia (45.2%) and Uganda (39.7%) had the highest proportion of households with no handwashing facilities (Table 3). For most of the other countries with available data, households had limited hygiene facilities with a place for handwashing but without soap and water.

### 3.2. WASH Indicators and Child Length

In the unadjusted models (Model I), in all but two countries, models predicted that children living in households with safely managed water supply had higher LAZ compared to those drinking from surface water (Table 4). After adjusting for covariates (Model II), safely managed drinking water was associated significantly with increased LAZ only in Kenya (β = 0.13, R^2^ = 0.20) and Tanzania (β = 0.08, R^2^ = 0.24). The associations remained significant after adjustments by sanitation facilities and hygiene practices in model III. Compared to children living in households drinking mainly from surface water, children in Rwanda in the unimproved water supply ladder had lower LAZ after adjustments (β = −0.10, R^2^ = 0.23).

In most countries, better sanitation facilities were associated with higher LAZ (Table 5) in the unadjusted models. A significant increase in LAZ in adjusted models was predicted for children living in households with basic sanitation facilities in Ethiopia (β = 0.07, R^2^ = 0.21), Tanzania (β = 0.08, R^2^ = 0.24), and Uganda (β = 0.11, R^2^ = 0.22) compared to children living in households practicing open defecation. Moreover, the positive associations remained significant in the models adjusted for water supply and hygiene practices. In Kenya and Tanzania, adjusted models predicted that children with unimproved sanitation had lower LAZ than children living in households with no sanitation. These negative associations remained significant in model III.

In Ethiopia and Tanzania, children living in households with basic hygiene practices had significantly higher predicted LAZ than those living in households with no handwashing facilities in the unadjusted models (Table 6). However, the associations were no longer significant after adjustment for covariates.

## 4. Discussion

Child stunting rates were high in East Africa, ranging from 20.5% in Kenya to 45% in Burundi. A global study using data from 116 countries over a 42-year period reported that underlying determinants of child undernutrition such as access to safe water and sanitation, maternal education, and dietary quality were strong drivers of the observed stunting reduction [3]. Increasing access to safe sanitation and water, improving child care practices through women’s education and gender equality, and increasing food security were also identified as key priority areas to accelerate stunting reduction, especially in sub-Saharan Africa [3]. From our results, there is still a need to improve WASH conditions in East Africa despite the progress made during the past decade. Drinking water availability and quality is a high priority need as more than half of the households in the East African region did not have access to an improved source of water or had to walk at least 30 min for water. Water availability can also impact household hygiene practices, which may explain the fact that the majority of the households had handwashing facilities but no water or soap.

Except for Kenya and Tanzania, where safely managed water was associated significantly with higher LAZ, there was no difference between predicted z-scores of children living in households drinking from surface water, unimproved, limited, and basic water supplies versus safely managed water. Improvement of only the quality of drinking water source and access may not be sufficient to have a positive effect on child linear growth in many situations [18]. In addition to coming from an improved source, water needs to be available on premise without excessive time required for collection as well as being free from microbial contamination [20].

Ethiopian and Tanzanian children living in households with limited and basic sanitation facilities were predicted to have higher LAZ scores than those living in households practicing open defecation. The high open defecation rates in Ethiopia (36.4%) and in Tanzania (22.2%) may be the basis for this association. Spears (2013) reported that open defecation significantly decreased children’s LAZ in 140 low- and middle-income countries (LMICs) even after controlling for gross domestic product (GDP) and maternal height. These results are similar to those in other LMICs [11,12,25] where poor WASH conditions were associated with shorter child length.

Some evidence indicates that diarrhea and helminth infection due to inadequate WASH contribute to linear growth faltering [14,26]. The diarrhea pathway may then explain the association between poor sanitation and lower height in Uganda where diarrhea prevalence was relatively high (32%). However, not as many Ethiopian (16.5%) and Tanzanian (16.2%) children were reported to have diarrhea. Moreover, cumulative diarrhea burden of more than five episodes before a child was 24 months old only explained 25% of the stunted growth in a pooled multi-country analysis [14]. Perhaps rapid catch-up growth between diarrhea episodes reduces its contribution to stunting [27].

Currently, EED has been a focus as a primary pathway by which inadequate WASH leads to stunting [10,15,28]. A cohort study conducted in Peru over 35 months showed that children living in households with poor sanitation and water quality were significantly one centimeter shorter than their counterparts with better conditions [12]. Furthermore, the effect was shown to be independent of diarrhea, suggesting that the decrease in linear growth might be due to a subclinical condition such as EED. Further analysis of our data showed that associations between better water quality and sanitation and higher LAZ scores remained significant after adjusting for diarrhea (Table S1). Thus, EED is another possible explanatory pathway for the role of suboptimal WASH on linear growth faltering in East African children. Poor WASH conditions lead to prolonged exposure to pathogens resulting in alteration of gut structure and function [29]. The changes in intestinal morphology, mainly atrophy of the villi and crypt elongation, result in a reduction in the capacity to absorb nutrients [29]. Decreased intestinal absorption will lead to nutrient deficiencies because the high nutritional needs of infants and young children will not be met, resulting in undernutrition. Additionally, there may be a loss of gut barrier function leading to the translocation of pathogenic agents. These pathogens trigger intestinal and systemic inflammation reactions that will divert nutrient utilization from growth [30]. Therefore, EED may at least partially explain the less than expected effectiveness of nutrition-specific interventions on stunting in areas with high burden of undernutrition and poor WASH indicators such as in the East African region.

In Rwanda, children living in households with unimproved water source were shorter compared to those with households drinking from surface water. Similarly, children living in households with unimproved sanitation facilities had lower LAZ compared to children with households practicing open defecation in Tanzania. These results may be explained by other factors in line with the multifactorial aspect of stunting. For example, the proportion of children meeting the minimum dietary diversity was the lowest (17.2%) among the children in households drinking from unimproved water JMP ladder compared to the other categories (data not shown). Additionally, the DHS data only provide information on the presence and types of sanitation facilities and whether they were shared with other households, but not if the facilities were being used. Furthermore, the safely managed sanitation category from the JMP sanitation ladder was not included in the analyses due to missing data on safe removal of child excreta from some countries. Thus, the variables used in our study could miss important aspects of the sanitation component of the JMP.

Our results add to the evidence that low-cost basic WASH interventions likely will not be sufficient to prevent the negative effects of suboptimal WASH on linear growth. In most cases, only the highest categories on ladders of water supply and sanitation facilities predicted significantly higher LAZ. Major improvements in WASH conditions are critically needed in each of the countries in East Africa, not only to observe the desired improvements in children’s anthropometrics, but also to reduce the morbidity prevalence for conditions such as diarrhea.

In addition to the safety and accessibility of the water supply already captured by the JMP indicators, other aspects of water quality need to be considered. Safely managed water may be intermittently unavailable to the households at various times, possibly leading to the use of undesirable water sources. Indicators such as the household water insecurity scale (HWISE) incorporate reliability and adequacy of water supply [31,32]. Water insecurity, as measured by the HWISE, has been associated with food insecurity in a multi-country study [33].

To optimize the potential of better sanitation in reducing fecal pathogen contamination, not only improved facilities are needed for each household, but also human feces need to be discarded safely. In addition, as exposure to animal feces has been associated with lower LAZ in children under 2 years old, such fecal exposure needs to be limited as much as possible [34,35]. Reduced exposure can be achieved through sanitation interventions at the community level with strong behavior change components. High adherence and safe removal of child feces was achieved in Bangladesh [36], Kenya [37], and Zimbabwe [38] with the provision of materials (scoops, child potty, and improved latrines) combined with frequent home visits by trained hygiene promoters.

In the present study, hygiene indicators were not associated with child length after adjustment with covariates. These results should not challenge the importance of optimal WASH practices in human health in general [18]. Hygiene practices could not be assessed comprehensively from the available data. The JMP indicators on hygiene only capture the presence of a handwashing station with water and soap, but do not include whether household members frequently and adequately wash their hands to reduce contamination. Collecting and integrating data on handwashing would likely improve assessment of hygiene practices.

The associations between water, sanitation, and hygiene conditions and child length differed by country. Better water quality and availability were important to increased child LAZ in Kenya and Tanzania because the associations remained significant in the models adjusted with sanitation in Kenya and with adjustment of sanitation and hygiene indicators in Tanzania. Kenya (18.1%) and Tanzania (14%) had the highest proportions of households drinking from surface water in East Africa. For Ethiopia, Tanzania and Uganda, ensuring households have improved latrines without sharing with other households appears to be an important predictor for child linear growth. In addition to having the highest proportions of open defecation in East Africa, Ethiopia (45.7%) and Tanzania (78.7%) had among the lowest rates of safe removal of child feces (data not shown).

Thus, because water, sanitation, or hygiene conditions influence child length differently for each country, the priorities for improvement must also be based on the local context. The importance of local context is the major reason that a meta-analysis was not conducted on these data in the present study. With the data from each country analyzed separately, we are able to provide evidence that is more context-specific on the importance of water, sanitation, and hygiene conditions for growth of young children.

Improving water sources, reducing open defecation, and increasing handwashing with soap should be priorities for each country. Success will demand high infrastructure coverage with appropriate and efficient behavioral change components. The Integrated Behavioral Model for Water, Sanitation, and Hygiene (IBM-WASH) integrates local contextual, psychosocial, and technological factors across multiple levels that may influence WASH behaviors in low income countries [39]. Most importantly, sustainability of such projects will require buy-in from the local communities and the various stakeholders. Collaboration of different public and private sectors working in nutrition, public health, agriculture, and education on integrated policies and programs also may give improved and sustained results. Future studies should test the effectiveness of safely managed water supplies and sanitation facilities, and appropriate handwashing practices to improve children’s nutritional status in areas with both high rates of child undernutrition and poor WASH conditions. Because these factors contribute to child growth through different pathways, they must be addressed concurrently to maximize intervention effectiveness.

Though this multi-country study is, to the best of our knowledge, the first in East Africa, some limitations must be acknowledged. First, data were obtained from cross-sectional surveys and therefore, no causality could be inferred from the results. Missing data on handwashing practices did not allow for modeling of the association between hygiene and child growth in Kenya, Rwanda, and Zambia. Moreover, all the limitations associated with survey-based studies should be recognized such as social desirability and recall biases. However, the DHS is widely accepted as high quality nationally representative data and the JMP estimates of WASH indicators for each country are based on these datasets.

## 5. Conclusions

Even though WASH indicators were noticeably different across East African countries, better WASH conditions generally were associated with improved child length. The influence of water and sanitation on linear growth varied across countries. Certain WASH indicators were more important than others for different countries. This study emphasizes the importance of environmental conditions on child nutritional status during the first 1000 days. Priority needs to be given to more comprehensive and context-specific WASH interventions where child stunting rates are persistently high.

## Figures and Tables

**Table 1:** Detailed sample size of the households with 6–23 mo children included for the eight East African countries.

Country	Burundi	Ethiopia	Kenya	Malawi	Rwanda	Tanzania	Uganda	Zambia
Original datasets	6493	10,937	21,718	6102	3926	10,898	5561	13,939
Datasets with observations for WASH variables	4536	5082	6641	3423	2511	4341	2641	5575
Final datasets (observations without anthropometric measurements deleted)	2226	2451	3978	1631	1177	2770	1370	2963

**Table 2:** WHO and UNICEF Joint Monitoring Programme for Water Supply, Sanitation and Hygiene (JMP) criteria (www.washdata.org).

Ladder	Characteristics
WATER	
Surface water	River, dam, lake, pond, stream, canal, or irrigation canal
Unimproved	Unprotected dug well or unprotected spring
Limited	Improved source ^1^ and collection time exceeds 30 min
Basic	Improved source ^1^ and collection time is no more than 30 min for roundtrip
Safely managed	Improved source ^1^ and available on premises and available when needed and free from fecal and chemical contamination ^2^
SANITATION	
Open defecation	Disposal of human feces in fields, forests, bushes, open bodies of water, beaches and other open spaces, or with solid waste
Unimproved	Pit latrines without a slab, hanging latrines, or bucket latrines
Limited	Improved facilities ^3^ and shared between two or more households
Basic	Improved facilities ^3^ and not shared with other households
Safely managed (not included in analyses in this paper)	Improved facilities ^3^ and not shared with other households and extra excreta are safely disposed in situ or transported and treated off-site
HYGIENE	
No facility	No handwashing facility on premises
Limited	Handwashing facility on premises without soap and water
Basic	Handwashing facility on premises with soap and water

^1^ Improved sources of drinking water include piped water, tube well, borehole, protected spring or protected well, rainwater, tanker truck, cart with small tank, or bottled water. ^2^ Data on fecal and chemical contamination were not available. ^3^ Improved sanitation facilities include flush/pour flush, piped sewer system, septic tanks, pit latrines, ventilated pit latrines, composting toilets, or pit latrines with slab.

**Table 3:** Characteristics of the study populations from their most recent Demographic and Health Survey (DHS): Burundi (2016–2017), Ethiopia (2016), Kenya (2015), Malawi (2017), Rwanda (2017), Tanzania (2017), Uganda (2016), and Zambia (2013–2014).

	Burundi		Ethiopia		Kenya		Malawi		Rwanda		Tanzania		Uganda		Zambia	
N	2226		2451		3978		1631		1177		2770		1370		2963	
	*n*	%	*n*	%	*n*	%	*n*	%	*n*	%	*n*	%	*n*	%	*n*	%
**Child Characteristics**																
Sex																
Male	1113	50.0	1150	46.9	1935	48.6	811	49.7	578	49.1	1387	50.1	691	50.4	1462	49.3
Female	1112	50.0	1301	53.1	2043	51.4	820	50.3	599	50.9	1383	49.9	679	49.6	1501	50.7
Stunting	1002	45.0	697	28.4	815	20.5	509	31.2	351	29.8	764	27.6	348	25.4	1068	36.1
Mean LAZ (SD)	−1.8 (1.2)		−0.8 (1.7)		−0.9 (1.4)		−1.2 (1.3)		−1.2 (1.5)		−1.2 (1.4)		−1.0 (1.5)		−1.3 (1.6)	
Stunting rates	1002	45.0	697	28.4	815	20.5	509	31.2	351	29.8	764	27.6	348	25.4	1068	36.1
Diarrhea in the past 2 weeks	685	31.0	405	16.5	864	21.7	491	30.1	203	17.2	449	16.2	438	32.0	629	21.2
Currently breastfed	2084	93.6	2261	92.2	1820	45.7	1433	87.4	1138	96.7	2483	89.6	1144	83.5	2461	89.1
**Maternal and Household Characteristics**																
Highest education level																
No education	975	43.8	1483	60.5	423	10.6	181	11.1	137	11.6	537	19.4	130	9.5	321	10.8
primary	970	43.6	759	31.0	2150	54.0	1086	66.6	854	72.6	1765	63.7	810	59.1	1162	54.5
Secondary	271	12.1	151	6.2	1022	25.7	332	20.3	160	13.6	438	15.8	334	24.4	936	31.6
Higher	9	0.4	58	2.3	383	9.6	32	1.9	26	2.2	30	1.1	96	7.0	90	3.0
Wealth index																
Poorest	461	20.7	862	35.2	936	23.5	401	24.6	306	26.0	676	14.4	284	20.7	756	25.5
Poor	491	22.1	419	17.1	698	17.5	388	23.8	238	20.2	588	21.2	276	20.1	715	24.1
Middle	474	21.3	359	14.6	669	16.8	330	20.2	224	19.1	513	18.5	272	20.0	633	21.4
Wealthier	435	19.6	317	12.9	704	17.7	261	16.0	202	17.1	518	18.7	236	17.2	521	17.6
Wealthiest	365	16.4	494	20.2	970	24.4	251	15.4	207	17.6	475	17.1	301	22.0	338	11.4
Area of residence																
Urban	200	9.0	454	18.5	1622	40.8	216	13.2	205	17.4	759	27.4	295	21.5	896	30.2
Rural	2026	91.0	1997	81.5	2356	59.2	1415	86.8	972	82.6	2011	72.6	1075	78.5	2067	69.7
**WASH Indicators (JMP ladder)**																
Drinking water																
Surface water	116	5.3	272	11.1	702	18.1	51	3.1	123	10.4	388	14.0	129	9.5	358	12.2
Unimproved	296	13.2	769	31.4	411	10.6	187	11.6	193	16.4	750	27.1	164	12.0	885	30.3
Limited	726	30.4	628	25.7	552	14.2	593	36.6	392	33.3	546	19.8	566	41.6	361	12.4
Basic	917	40.7	503	20.6	953	24.5	593	36.6	355	30.2	472	17.1	305	22.4	877	30.0
Safely managed	170	10.4	273	11.2	1268	32.6	195	12.0	112	9.6	608	22.0	197	14.5	442	15.1
Missing	1		6		91		12		1		6		9		40	
Sanitation																
Open defecation	49	2.2	890	36.4	605	15.3	94	5.6	42	3.5	354	22.2	111	8.1	625	21.1
Unimproved	1003	45.0	1334	54.5	1388	35.0	211	12.9	303	25.8	484	30.3	724	53.1	1174	39.7
Limited	227	10.2	119	4.85	1174	29.6	528	32.4	209	17.8	338	21.2	253	18.5	476	16.1
Basic	947	42.5	103	4.2	793	20.0	798	48.9	620	52.8	419	26.3	277	20.3	683	23.1
Missing	0	0	5		17				3		1175		5		5	
Hygiene																
No handwashing facility	20	0.9	1105	45.2	30	4.8	266	16.4	8	6.4	464	16.7	541	39.7	43	4.1
Limited	2084	93.6	1180	48.3	351	57.0	1195	73.7	82	65.4	1028	37.1	468	34.3	732	70.0
Basic	121	5.5	158	6.4	235	38.1	161	9.9	35	28.2	1278	46.1	355	26.0	271	25.9
Missing	1		8		3362		9		1051				6		1917	

WHO and UNICEF Joint Monitoring Programme (JMP) for water supply, sanitation and hygiene (See Table 2). Standard deviation (SD). Length-for-age z-scores (LAZ)

**Table 4:** Predicted increments in young child length-for-age z-scores (LAZ) (6–23 mo) associated with JMP water supply indicators in East Africa.

Countries	JMP Ladder	Model I	Model II	R^2^	Model III	R^2^
Burundi	Unimproved	−0.03	−0.06	0.25	−0.06	0.25
	Limited	0.01	−0.04		−0.04	
	Basic	0.03	−0.02		−0.02	
	Safely managed	0.19 ***	0.06		0.07	
Ethiopia	Unimproved	−0.02	0.01	0.21	0.01	0.22
	Limited	−0.05	−0.02		−0.01	
	Basic	−0.08	−0.06		−0.05	
	Safely managed	0.70	0.05		0.05	
Kenya	Unimproved	0.04	0.01	0.20	0.01	0.21
	Limited	0.05 *	0.02		0.03	
	Basic	0.07 **	0.01		0.02	
	Safely managed	0.18 ***	0.13 **		0.13 **	
Malawi	Unimproved	0.04	0.03	0.09	0.03	0.10
	Limited	0.11	0.07		0.08	
	Basic	0.06	0.02		0.03	
	Safely managed	0.12 *	0.04		0.05	
Rwanda	Unimproved	−0.11 *	−0.10 **	0.23	−0.10 **	0.23
	Limited	−0.07	−0.05		−0.05	
	Basic	−0.02	−0.02		−0.02	
	Safely managed	0.07	0.01		0.01	
Tanzania	Unimproved	0.03	0.02	0.24	0.03	0.24
	Limited	0.11	−0.02		−0.01	
	Basic	0.06	0.02		0.02	
	Safely managed	0.14 ***	0.08 *		0.07 *	
Uganda	Unimproved	0.05	0.03	0.22	0.03	0.22
	Limited	0.10	0.03		0.02	
	Basic	0.04	0.01		0.01	
	Safely managed	0.10*	0.02		0.01	
Zambia	Unimproved	0.01	-0.01	0.20	0.01	0.20
	Limited	0.04	0.03		0.03	
	Basic	0.04	0.01		0.01	
	Safely managed	0.13 ***	0.05		0.05	

Results are expressed as standardized beta coefficients. WHO and UNICEF Joint Monitoring Programme (JMP) for water supply, sanitation and hygiene (See Table 2). Reference: surface water. R^2^: adjusted coefficient of determination. Model I: unadjusted; Model II: adjusted for child sex, age, and breastfeeding status, for maternal highest level of education, age, and height, and for household wealth index and area of residence (urban vs. rural); Model III: additionally adjusted for other WASH indicators. * *p* < 0.05, ** *p* < 0.01, *** *p* < 0.001.

**Table 5:** Predicted increments in young child LAZ (6–23 mo) associated with JMP sanitation indicators in East Africa.

Countries	JMP Ladder	Model I	Model II	R^2^	Model III	R^2^
Burundi	Unimproved	0.11	0.05	0.24	0.06	0.25
	Limited	0.17 ***	0.05		0.05	
	Basic	0.20 **	0.08		0.08	
Ethiopia	Unimproved	0.06	0.08 **	0.21	0.09 **	0.22
	Limited	0.06 *	0.07 **		0.05*	
	Basic	0.08 ***	0.07 **		0.06 **	
Kenya	Unimproved	0.05 *	−0.08 *	0.20	−0.08 *	0.21
	Limited	0.14 ***	−0.01		−0.01	
	Basic	0.13 ***	−0.01		−0.01	
Malawi	Unimproved	0.03	0.02	0.09	0.03	0.10
	Limited	0.02	−0.04		−0.04	
	Basic	0.02	−0.02		−0.02	
Rwanda	Unimproved	−0.02	−0.02	0.23	−0.02	0.23
	Limited	0.03	−0.08		−0.07	
	Basic	0.06	−0.05		−0.04	
Tanzania	Unimproved	−0.04	−0.04 *	0.24	−0.04 *	0.24
	Limited	0.09 ***	0.07 *		0.06 *	
	Basic	0.11 ***	0.08 **		0.06 *	
Uganda	Unimproved	0.01	0.03	0.22	0.03	0.22
	Limited	0.08	0.09		0.10	
	Basic	0.12 *	0.11 *		0.11 *	
Zambia	Unimproved	0.04	0.02	0.20	0.02	0.20
	Limited	0.08 **	0.04		0.04	
	Basic	0.06	0.02		0.02	

Results are expressed as standardized beta coefficients. Reference: open defecation. WHO and UNICEF Joint Monitoring Programme (JMP) for water supply, sanitation and hygiene. R^2^: adjusted coefficient of determination. Model I: unadjusted; Model II: adjusted for child sex, age, and breastfeeding status, for maternal highest level of education, age, and height, and for household wealth index and area of residence (urban vs. rural); Model III: additionally adjusted for other WASH indicators. * *p* < 0.05, ** *p* < 0.01, *** *p* < 0.001.

**Table 6:** Predicted increments in young child LAZ (6–23 mo) associated with JMP hygiene indicators in East Africa.

Countries	JMP Ladder	Model I	Model II	R^2^	Model III	R^2^
Burundi	Limited	−0.19	−0.02	0.24	−0.02	0.25
	Basic	0.41	−0.03		−0.04	
Ethiopia	Limited	−0.03	−0.03	0.21	−0.03	0.25
	Basic	0.08 *	0.05		0.04	
Malawi	Limited	0.04	0.02	0.09	0.03	0.09
	Basic	0.07	0.05		0.05	
Tanzania	Limited	−0.01	−0.02	0.23	−0.02	0.24
	Basic	0.08 ***	0.03		0.03	
Uganda	Limited	0.04	−0.01	0.22	−0.01	0.22
	Basic	0.05	0.01		0.01	

Results are expressed as standardized beta coefficients. WHO and UNICEF Joint Monitoring Programme (JMP) for water supply, sanitation and hygiene. Reference: no handwashing facilities. R^2^: adjusted coefficient of determination. Model I: unadjusted; Model II: adjusted for child sex, age, and breastfeeding status, for maternal highest level of education, age, and height, and for household wealth index and area of residence (urban vs. rural); Model III: additionally adjusted for other WASH indicators. * *p* < 0.05, ** *p* < 0.01, *** *p* < 0.001.

## Supplementary Materials

The following are available online at https://www.mdpi.com/1660-4601/17/17/6262/s1, Table S1: Increments in young child LAZ (6–23 mo) predicted by JMP water supply indicators and frequent diarrheal episodes in East Africa.
