# Supplementary material for: The WHO and UNICEF Joint Monitoring Programme (JMP) Indicators for Water Supply, Sanitation and Hygiene and Their Association with Linear Growth in Children 6 to 23 Months in East Africa
Source: Int J Environ Res Public Health. 2020 Aug 28;17(17):6262. doi: 10.3390/ijerph17176262 (PMC7503684; doi:10.3390/ijerph17176262)
Supplement: Supplementary file 1 [file ijerph-17-06262-s001.pdf]

Table S1.

Increments in young child LAZ (6 – 23 mo) predicted by JMP water supply indicators and frequent diarrheal episodes in East Africa.

**JMP ladder**	**Burundi**	**Ethiopia**	**Kenya**	**Malawi**	**Rwanda**	**Tanzania**	**Uganda**	**Zambia**
Water supply (Ref. Surface water)								
R^2	0.25	0.21	0.20	0.09	0.23	0.24	0.22	0.20
Unimproved	-0.06	0.01	0.01	0.03	-0.10**	0.02	0.03	0.01
Limited	-0.04	-0.02	0.02	0.07	-0.05	-0.02	0.03	0.03
Basic	-0.02	-0.06	0.01	0.02	-0.02	0.02	0.02	0.02
Safely managed	0.06	0.05	0.13**	0.04	0.01	0.08*	0.02	0.02
Sanitation facilities (Ref. Open defecation)								
R^2	0.24	0.22	0.20	0.09	0.23	0.24	0.22	0.20
Unimproved	0.06	0.08**	-0.08*	0.02	-0.03	-0.04*	0.03	0.01
Limited	0.05	0.07**	-0.01	-0.04	-0.08	0.07*	0.09	0.04
Basic	0.08	0.07**	-0.01	-0.02	-0.05	0.08**	0.11*	0.02
Hygiene practices (Ref. No handwashing facilities								
R^2	0.24	0.21	-	0.09	-	0.23	0.22	-
Limited	-0.02	-0.03	-	0.02	-	-0.02	-0.01	-
Basic	-0.03	0.05	-	0.05	-	0.03	0.01	-

Results are expressed as standardized beta coefficients.

JMP: WHO and UNICEF Joint Monitoring Programme for Water Supply, Sanitation and Hygiene (See table 2). R^2: adjusted coefficient of determination. Models were adjusted for child sex, age, breastfeeding status, and diarrhea during the last two weeks, for maternal highest level of education, age, and height, and for household wealth index and area of residence (urban vs. rural), and for other WASH indicators. *p<0.05, **p<0.01, ***p<0.001.
